# Port retrieval for salvage of tissue expansion in case of lost or malfunctioning port

**DOI:** 10.4103/0970-0358.73441

**Published:** 2010

**Authors:** Lalit K. Makhija, Manoj K. Jha, Sameek Bhattacharya, Ashish Rai, Sharad Mishra, Anjubala Dey

**Affiliations:** Department of Plastic and Reconstructive Surgery, PGIMER & Dr. RML Hospital, New Delhi -110 001, India

**Keywords:** Port complications, port retrieval, tissue expansion

## Abstract

Tissue expansion though a promising modality of reconstructive surgery is fraught with many complications. In addition to expander-related complications, subcutaneous port-related mishaps during tissue expansion, though infrequent, can result in procedure failures. We are reporting two patients with port-related complications. In one patient, there was failure to localise the port and the other had a leaking port. Both the expanders were salvaged by retrieving the ports. In the former, as the port was competent, it was simply exteriorised. But in the later case, the connecting tube was retrieved and the incompetent port was replaced with a Luer lock external port. Both the cases were successfully salvaged without any further complications. Expansions were completed and requisite reconstructive end points were achieved.

## INTRODUCTION

In tissue expansion, in addition to those complications related to the expanding silicone envelope, injection port has also been associated with some specific complications like exposure, lost port, port malfunction, dislocation and rotation. Instead of giving up on these expanders and loosing time and money or adding further morbidity, we feel that it is worth salvaging them without adding ay risk to the patients by addressing the port specific problems.[1]

## CASE REPORTS

### Case 1

**Figure 1:**
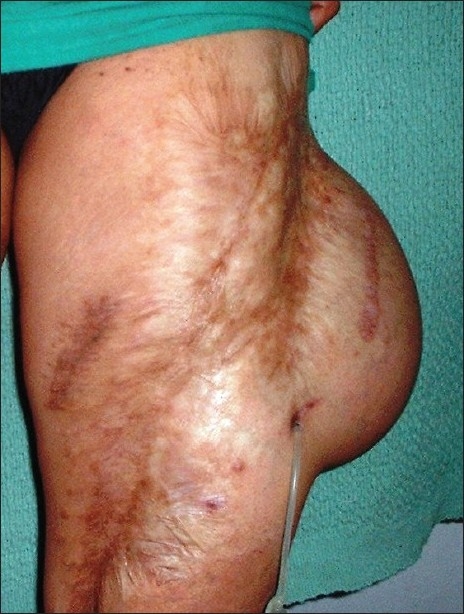
Case 1: Thigh scar; patient with exteriorised port after completion of expansion

*The “lost” port*: A tissue expander with subcutaneous port was placed in the thigh for management of post traumatic scars. Later, we failed to locate the port owing to the thick subcutaneous tissue. Although we injected saline after localising the port by ultrasound, every time this exercise was not logistically feasible. Hence, we exteriorised the subcutaneous port after ultrasongraphic localisation. Tube site was dressed regularly and expander inflation was continued. The patient underwent expansion and reconstruction successfully [Figure 1].[2]

### Case 2

**Figure 2:**
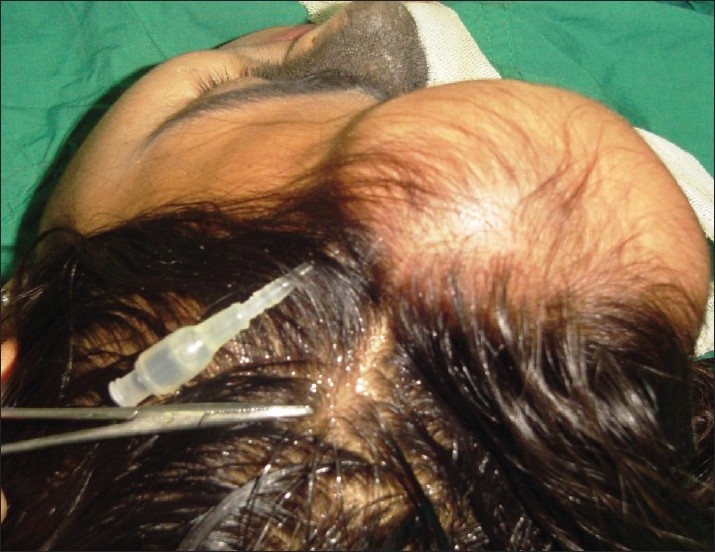
Case 2: Exteriorised tube with Luer lock port with abandoned subcutaneous port *in situ*

*The “malfunctioning” port*: Tissue expanders were placed in cheek and forehead in a 15-year-old girl with giant congenital melanocytic nevus of face. After uneventful expansion for about 2 months, the port became incompetent leading to slow deflation of the expander following saline injection. In order to salvage the expander, the connecting tube was retrieved by giving a small incision leaving the original port *in situ* and an external “Luer” lock port was connected to the retrieved tubing [Figure 2]. Expansion with external port was continued with strict aseptic precautions. Reconstruction was completed successfully without any other untoward incident.

## DISCUSSION

**Figure 3:**
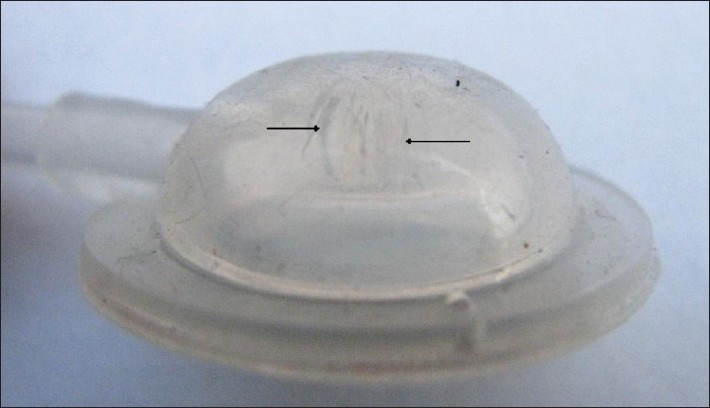
Magnified view of the “leaking” port; multiple linear tears on the port cover

Overall expander related complication rates in the literature range from 5 to 30%.[3–5] Such complications usually necessitate abandonment or modification of procedure. Numerous complications have also been associated with subcutaneous ports, including seroma, hematoma, skin erosion, port dislocation and port leakage.[6–8] Cunha *et al*. reported a port failure rate of 5.4% in their 10-year experience.[5] Port incompetence can be attributed to various factors. Multiple punctures of the port septum over a long period, especially with a wide bore needle, may cause the septum to wear out or dislodge small particles of the seal. Sometimes, if too much force is employed while introducing the needle in the port, the needle tip may bend by striking the metal base of the port and the hooked needle may tear the seal at time of withdrawal. This was probably the cause of port failure in the second case. We found multiple linear tears in the port on examination under magnification [Figure 3].

There are few reports regarding use of external ports for tissue expansion.[6–10] Complication rates of primary external port placement range from 5.6 to 23%.[6–9] In our patients, there was no expander infection after port exteriorisation. This could be due to the fact that body of expander and port tube assembly were in separate capsules at the time of exteriorisation and therefore we did not breach the expander pocket while exteriorising the port. Additionally, strict asepsis was also followed during exteriorisation and subsequent expansion.

There is a paucity of literature with regard to expansion salvage in face of lost or incompetent port. There are anecdotal references of ultrasonic localisation of a lost port,[11] but there is no report of port exteriorisation as solution. By this simple technique of port retrieval and replacement, precious expansion can be salvaged. Moreover, the expansion phase is not prolonged and added patient morbidity is obviated.
